# Expertenzertifikat: „*Otologische‑, Neurootologische- und (laterale) Schädelbasis-Chirurgie*“

**DOI:** 10.1007/s00106-026-01767-9

**Published:** 2026-05-06

**Authors:** Hubert Löwenheim, Thomas Deitmer, Dirk Beutner, Thomas Zahnert, Timo Stöver, Martin Jäckel, Christian Betz, Andreas Neumann, Christoph Arnoldner, Thomas K. Hoffmann

**Affiliations:** 1 Klinik für Hals‑, Nasen‑, Ohrenheilkunde, Universitätsklinikum Tübingen, Universität Tübingen, Elfriede-Aulhorn-Strasse 5, 72076 Tübingen, Deutschland; 2 Kopf- und Hals-Chirurgie e. V., Deutsche Gesellschaft für Hals-Nasen-Ohren-Heilkunde, Friedrich-Wilhelm-Str. 2, 53113 Bonn, Deutschland; 3 Klinik für Hals‑, Nasen‑, Ohrenheilkunde, Kopf- und Halschirurgie, Universitätsklinikum Göttingen, Göttingen, Deutschland; 4 Klinik für Hals‑, Nasen‑, Ohrenheilkunde, Kopf- und Halschirurgie, Universitätsklinikum Dresden, Dresden, Deutschland; 5 Klinik für Hals‑, Nasen‑, Ohrenheilkunde, Universitätsmedizin Frankfurt a.M., Goethe-Universität Frankfurt a.M., Theodor-Stern-Kai 7, 60590 Frankfurt a.M, Deutschland; 6 Klinik für Hals‑, Nasen‑, Ohrenheilkunde, Helios Kliniken Schwerin, Wismarsche Straße 393-397, 19055 Schwerin, Deutschland; 7 Klinik für Hals‑, Nasen‑, Ohrenheilkunde, Kopf- und Halschirurgie, Universitätsklinikum Hamburg-Eppendorf, Martinistr. 52, 20246 Hamburg, Deutschland; 8 Klinik für Hals‑, Nasen‑, Ohrenheilkunde, Rheinland Klinikum Neuss GmbH, Preußenstraße 84, 41464 Neuss, Deutschland; 9 Universitätsklinik für Hals‑, Nasen- und Ohrenheilkunde, Kopf- und Halschirurgie, Medizinische Universität Wien, Wien, Österreich; 10 Klinik für Hals‑, Nasen‑, Ohrenheilkunde, Kopf- und Halschirurgie, Universitätsklinikum Ulm, Frauensteige 12, 89070 Ulm, Deutschland

**Keywords:** HNO, Personenzertifizierung, Kopf-Hals-Chiurgie, Logbuch, Otochirurgie, Schädelbasischirurgie, ORL, Expert certification, Head neck surgery, Catalogue, Otosurgery, Skull base surgery

## Abstract

Die Deutsche Gesellschaft für Hals-Nasen-Ohren-Heilkunde, Kopf- und Hals-Chirurgie e. V. (DGHNO-KHC) und die Deutsche Akademie für Hals-Nasen-Ohren-Heilkunde, Kopf- und Hals-Chirurgie e. V. (DAHNO-KHC) haben unter Mitwirkung der entsprechenden Arbeitsgemeinschaften *Expertenzertifikate* für die Teilbereiche „Kopf-Hals-Onkochirurgie“ und „Nasennebenhöhlen- und (anteriore) Schädelbasis-Chirurgie“ entwickelt. Ziel war es, in Analogie zu internationalen Standards die Expertise der Antragstellenden für den jeweiligen Teilbereich im Sinne der Personenzertifizierung darzustellen. Für den Teilbereich der chirurgischen Otologie, Neurootologie und lateralen Schädelbasis wurde nun durch die Arbeitsgemeinschaft Deutschsprachiger Audiologen, Neurootologen und Otologen (ADANO) ein Kriterienkatalog erarbeitet, dessen Erfüllung eine Person für den Erwerb des Expertenzertifikats „Otologische‑, Neurootologische- und (laterale) Schädelbasis-Chirurgie“ qualifiziert. Das Personen-Zertifikat kann als Nachweis der individuellen Expertise verwendet werden. Die praktische Umsetzung erfolgt durch eine unabhängige Zertifizierungsstelle (ClarCert GmbH) im Auftrag der DGHNO-KHC und unter Mitarbeit der DAHNO-KHC sowie der jeweiligen Arbeitsgemeinschaften. Anträge können ab sofort von Mitgliedern der DGHNO-KHC oder der DAHNO-KHC für das Expertenzertifikat „Otologische‑, Neurootologische- und (laterale) Schädelbasis-Chirurgie“ gestellt werden.

**Zusatzmaterial online:**

In der Online-Version dieses Beitrags (10.1007/s00106-026-01767-9) steht das Logbuch für das Expertenzertifikat „Otologische‑, Neurootologische- und (laterale) Schädelbasis-Chirurgie“ als Begleitmaterial zum Download zur Verfügung.

## Abstract (en)

The German Society of Otorhinolaryngology, Head and Neck Surgery (DGHNO-KHC) and the German Academy of Otorhinolaryngology, Head and Neck Surgery (DAHNO-KHC), in cooperation with the relevant working groups, have developed expert certificates for the subfields “head and neck onco-surgery” and “paranasal sinus and (anterior) skull base surgery.” The aim was to demonstrate the applicants’ expertise in the respective subfield, in line with international standards. For the subspecialty “surgical otology, neurotology, and lateral skull base surgery,” the Association of German-Speaking Audiologists, Neurotologists, and Otologists (ADANO) has developed a set of criteria for obtaining the expert certificate “Otological, neurotological, and (lateral) skull base surgery.” This personal certificate can be used to demonstrate individual expertise. Practical implementation is carried out by an independent certification body (ClarCert GmbH) on behalf of the DGHNO-KHC and in cooperation with the DAHNO-KHC and the respective working groups. Applications for the expert certificate can now be submitted by members of the DGHNO-KHC and the DAHNO-KHC.

Das Fachgebiet der Hals-Nasen-Ohren-Heilkunde, Kopf- und Hals-Chirurgie bildet konservative und breite operative Bereiche ab. Nach der Facharztausbildung findet häufig eine vertiefende chirurgische Subspezialisierung statt, die sich in der Vergangenheit u. a. in der Zusatzweiterbildung „Spezielle HNO-Chirurgie“ widerspiegelte. Die Bundesärztekammer hat diese mit der Reform der Weiterbildung (MWBO 2003) eingestellt und wird diese auf absehbare Zeit nicht wieder implementieren. Zur Abbildung der weiterführenden chirurgischen Qualifikation in der Onkologie und der Rhinologie wurde auf Initiative der Deutschen Gesellschaft für Hals-Nasen-Ohren-Heilkunde, Kopf- und Hals-Chirurgie e. V. (DGHNO-KHC) und der Deutschen Akademie für Hals-Nasen-Ohren-Heilkunde, Kopf- und Hals-Chirurgie e. V. (DAHNO-KHC) in Zusammenarbeit mit den fachspezifischen Arbeitsgemeinschaften ein Konzept zum Erwerb von Expertenzertifikaten etabliert. Die bestehenden Zertifikate zur „Kopf-Hals-Onko-Chirurgie“ [1] und zur „Nasennebenhöhlen- und (anteriore) Schädelbasis-Chirurgie“ [2] sollen nun durch den Teilbereich „Otologische‑, Neurootologische- und (laterale) Schädelbasis-Chirurgie“ ergänzt werden. Durch die Arbeitsgemeinschaft Deutschsprachiger Audiologen, Neurootologen und Otologen (ADANO) wurde ein Kriterienkatalog erarbeitet, der für den Erwerb des Expertenzertifikats „Otologische‑, Neurootologische- und Schädelbasis-Chirurgie“ qualifiziert. Die Arbeitsgemeinschaft Schädelbasis- und kraniofaziale Chirurgie der DGHNO-KHC wurde eingebunden und hat den Katalog konsentiert. Hierfür werden entsprechende Inhalte in einem Logbuch hinterlegt. Ziel ist es, die persönliche Expertise der Antragstellenden für den Teilbereich „Otologische‑, Neurootologische- und (laterale) Schädelbasis-Chirurgie“ darzustellen. Dies erfolgt in Analogie zu internationalen Standards. Das Zertifikat kann zum Nachweis der individuellen Expertise genutzt werden, z. B. bei anderen Zertifizierungsverfahren (bspw. CI-versorgende Einrichtung/CIVE). Die praktische Umsetzung erfolgt durch eine unabhängige Zertifizierungsstelle (ClarCert GmbH) im Auftrag der DGHNO-KHC unter Mitarbeit der DAHNO-KHC sowie der Arbeitsgemeinschaft ADANO. Anträge können ab sofort von Mitgliedern der DGHNO-KHC oder der DAHNO-KHC für das Expertenzertifikat „Otologische‑, Neurootologische- und (laterale) Schädelbasis-Chirurgie“ gestellt werden.

## Kriterien

Voraussetzung für den Erwerb des Zertifikats ist die Erfüllung der nachfolgend genannten Inhalte:

- *Mitgliedschaft* bei der DGHNO-KHC
- *Facharztqualifikation* für Hals-Nasen-Ohren-Heilkunde (**Urkunde**)
- *Handlungskompetenzen* wie folgt (**Logbuch**; bei Operationen als Erstoperateur):
  - Diagnostik von (neuro)otologischen Erkrankungen
  - 50 operative Eingriffe bei Erkrankungen des äußeren Gehörgangs (Exostosen, Cholesteatom, Tumoren, Stenosen, Atresien)
  - 100 operative Eingriffe bei chronischen entzündlichen otologischen Erkrankungen (chronische Otitis media mesotympanalis und epitympanalis, davon min. 25 mit mastoidaler Beteiligung und min. 25 mit Ossikelrekonstruktion)
  - 25 Mastoidektomien bei akuten entzündlichen otologischen Erkrankungen (davon min. 10 pädiatrische Eingriffe)
  - 25 diagnostische und therapeutische Tympanoskopien
  - 25 operative Eingriffe bei Otosklerose (Stapesplastik, Stapesrevisionen)
  - 5 operative Eingriffe bei Glomus tympanicum (Typ A/B) oder anderen gutartigen Mittelohrtumoren
  - 10 neurootologische Eingriffe am Labyrinth (z. B. Dehiszenzen, Labyrinthfisteln, Dekompression des Saccus endolymphaticus)
  - 50 Cochleaimplantate (inklusive Revisionschirurgie und pädiatrischer Eingriffe)
  - 10 aktive Mittelohrimplantate und Knochenleitungsimplantate
  - 10 Eingriffe bei Tumoren im Felsenbein
  - 10 subtotale und totale Petrosektomien oder Schädelbasisrevisionen, z. B. bei Liquorrhö
  - 10 Behandlungen von operativen Komplikationen nach otologischen Behandlungen (z. B. Fazialisparesen, Liquorfisteln)
  - 5 Teilnahmen oder Mitgestaltungen an/von Fortbildungsveranstaltungen oder Studien zu Schwerpunktthemen (Otologie, Neurootologie, laterale Schädelbasis, z. B. zentrale oder dezentrale Kurse der Akademie, ADANO, Arbeitsgemeinschaft Schädelbasis- und kraniofaziale Chirurgie/ASKRA)

Die erbrachten Leistungen für die Qualifikation sind durch ein von der ärztlichen HNO-Klinikleitung (bzw. Vertretung, falls Leitung den Antrag selbst stellt) unterzeichnetes und gestempeltes Zeugnis entsprechend dem Logbuch (siehe Zusatzmaterial online) nachzuweisen. Durch die Unterschrift der HNO-ärztlichen Klinikleitung bzw. Vertretung bürgt der Unterzeichner für die Richtigkeit der vom Antragssteller gemachten Angaben. Die Überprüfung erfolgte persönlich und aufgrund aller im Logbuch geforderten Unterlagen auf Plausibilität und Vollständigkeit. Die vollständigen Nachweisunterlagen sind vom Antragssteller zu archivieren (z. B. Op.-Berichte und Teilnahme-Nachweise) und auf Nachfrage vorzulegen.

## Antragstellung

Das Antragsformular (Homepage ClarCert) ist mit Urkunden und dem Logbuch-Zeugnis digital (pdf-Dokumente) zu senden an:

info@clarcert.com

## Kosten

Zum Zeitpunkt der Drucklegung 225 € + Mehrwertsteuer; die Gültigkeit des Zertifikats ist zeitlich nicht begrenzt. Auf Wunsch kann eine Rezertifizierung im Sinne der Aktualisierung erfolgen.

## Perspektive

Der Zertifikatserwerb umfasst aktuell drei chirurgische Teilbereiche des Fachgebiets der Hals-Nasen-Ohren-Heilkunde, Kopf- und Hals-Chirurgie. Weitergehende Qualifikationen und vertiefende Subspezialisierungen in diesen Teilbereichen können durch nationale und internationale „Fellowships“ zusätzlich erworben werden. Entsprechende Empfehlungen hierzu befinden sich in Vorbereitung. Darüber hinaus ist geplant, konservative Teilbereiche bei der Personenzertifizierung abzudecken. Hierzu werden aktuell über die spezifischen Arbeitsgemeinschaften Vorschläge eingeholt.

## Supplementary Information

Logbuch für das Expertenzertifikat „Otologische‑, Neurootologische- und (laterale) Schädelbasis-Chirurgie“

## Data Availability

Daten können frei verfügbar gemacht werden.
