# Supplementary material for: Expert certificate: “Otological, neurotological, and (lateral) skull base surgery”
Source: HNO. 2026 May 6;74(7):465–7. [Article in German] doi: 10.1007/s00106-026-01767-9 (PMC13303761; doi:10.1007/s00106-026-01767-9)
Supplement: Supplementary file 1 — Logbuch für das Expertenzertifikat „Otologische‑, Neurootologische- und (laterale) Schädelbasis-Chirurgie“ [file 106_2026_1767_MOESM1_ESM.docx]

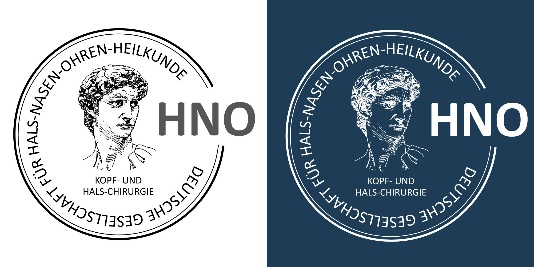

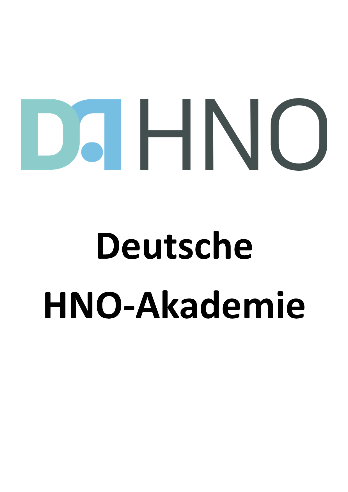


**Logbuch für das Expertenzertifikat**

**„Otologische-, Neurootologische- und (laterale) Schädelbasis-Chirurgie“**

Name:

Vorname:

Titel:

Geburtsdatum:

Facharzt-Urkunde für HNO-Heilkunde vom:

Dokumentation der Inhalte des Logbuches und der bisherigen beruflichen Tätigkeit als Arzt/Ärztin durch ein Zeugnis der Klinik-/Abteilungsleitung incl. Nennung der Ist-Zahl, Datum, Handzeichen pro Zeile.

| **Bezeichnung der Tätigkeit** | **Sollzahl** | **Ist-Zahl** | **Datum** | **Hdz.** |
| --- | --- | --- | --- | --- |
| Diagnostik von (neuro)otologischen Erkrankungen | keine | keine |  |  |
| Operative Eingriffe bei Erkrankungen des äußeren Gehörgangs (Exostosen, Cholesteatom, Tumoren, Stenosen, Atresien) | 50 |  |  |  |
| Operative Eingriffe bei chronischen entzündlichen otologischen Erkrankungen (chronische Otitis media mesotympanalis und epitympanalis)  - mit mastoidaler Beteiligung  - mit Ossikelrekonstruktion | 100  davon:  25  25 |  |  |  |
| Mastoidektomien bei akuten entzündlichen otologischen Erkrankungen  - pädiatrische Eingriffe | 25  davon:  10 |  |  |  |
| Diagnostische und therapeutische Tympanoskopien | 25 |  |  |  |
| Operative Eingriffe bei Otosklerose  (Stapesplastik, Stapesrevisionen) | 25 |  |  |  |
| Operative Eingriffe bei Glomus tympanicum (Typ A/B) oder anderen gutartigen Mittelohrtumoren | 5 |  |  |  |
| Neurootologische Eingriffe am Labyrinth (z.B. Dehiszenzen, Labyrinthfisteln, Dekompression des Saccus endolymphaticus) | 10 |  |  |  |
| Cochlea Implantate (inklusive Revisionschirurgie und pädiatrische Eingriffe) | 50 |  |  |  |
| Aktive Mittelohrimplantate und Knochenleitungsimplantate | 10 |  |  |  |
| Eingriffe bei Tumoren im Felsenbein | 10 |  |  |  |
| Subtotale und totale Petrosektomien oder Schädelbasisrevisionen z.B. bei Liquorrhoe | 10 |  |  |  |
| Behandlungen von operativen Komplikationen nach otologischen Behandlungen (z.B. Fazialisparese, Liquorfisteln) | 10 |  |  |  |
| Teilnahmen oder Mitgestaltung an/von Fortbildungsveranstaltungen oder Studien zu Schwerpunktthemen (Otologie, Neurootologie, laterale Schädelbasis z.B. zentrale oder dezentrale Kurse der Akademie, ADANO, ASKRA) | 5 |  |  |  |

Die erbrachten Leistungen wurden vom Antragsteller/Antragstellerin persönlich erbracht. Durch seine Unterschrift bürgt der Unterzeichner für die Richtigkeit der vom Antragssteller gemachten Angaben. Die Überprüfung erfolgte persönlich und aufgrund aller im Logbuch geforderten Unterlagen auf Plausibilität und Vollständigkeit.

Stempel, Datum und vollständige Anschrift sowie finale Unterschrift des ärztlichen Leiters / der Leiterin der HNO-Klinik (bzw. Vertretung).
